# Investigating sources for variability in volunteer kinematics in a braking maneuver, a sensitivity analysis with an active human body model

**DOI:** 10.3389/fbioe.2023.1203959

**Published:** 2023-10-16

**Authors:** Emma Larsson, Johan Iraeus, Johan Davidsson

**Affiliations:** Department of Mechanics and Maritime Sciences, Chalmers University of Technology, Gothenburg, Sweden

**Keywords:** active human body model, kinematics, sensitivity study, pre-crash, variability

## Abstract

Occupant kinematics during evasive maneuvers, such as crash avoidance braking or steering, varies within the population. Studies have tried to correlate the response to occupant characteristics such as sex, stature, age, and BMI, but these characteristics explain no or very little of the variation. Therefore, hypothesis have been made that the difference in occupant response stems from voluntary behavior. The aim of this study was to investigate the effect from other sources of variability: in neural delay, in passive stiffness of fat, muscle tissues and skin, in muscle size and in spinal alignment, as a first step towards explaining the variability seen among occupants in evasive maneuvers. A sensitivity analysis with simulations of the SAFER Human Body Model in braking was performed, and the displacements from the simulations were compared to those of volunteers. The results suggest that the head and torso kinematics were most sensitive to spinal alignment, followed by muscle size. For head and torso vertical displacements, the range in model kinematics was comparable to the range in volunteer kinematics. However, for forward displacements, the included parameters only explain some of the variability seen in the volunteer experiment. To conclude, the results indicate that the variation in volunteer vertical kinematics could be partly attributed to the variability in human characteristics analyzed in this study, while these cannot alone explain the variability in forward kinematics. The results can be used in future tuning of HBMs, and in future volunteer studies, when further investigating the potential causes of the large variability seen in occupant kinematics in evasive maneuvers.

## 1 Introduction

With the introduction of automated crash avoidance systems, such as automated emergency braking or evasive steering assist maneuvers, many vehicle crashes can be prevented or mitigated (Östling et al., 2019; Seacrist et al., 2020; Tan et al., 2020; Leledakis et al., 2021). While these system-induced maneuvers often reduce the crash severity or prevent the crash altogether, the maneuver can alter the occupant position or muscle activation (Ólafsdóttir et al., 2013; Kirschbichler et al., 2014; Holt et al., 2020), and consequently affect the injury outcome if the crash was not avoided (Bose et al., 2010; McMurry et al., 2018; Nie et al., 2018). As such, it is important to consider evasive maneuvering prior to a crash in evaluation of passive vehicle safety systems.

Human body models (HBMs) are used to evaluate vehicle occupant safety, and there are several models available, such as the SAFER HBM (Pipkorn et al., 2021), THUMS (Kato et al., 2018), GHBMC (Devane et al., 2019) and VIVA+ (John et al., 2022). Some of these models have been further developed by adding models of musculature with controlled activation, hereafter referred to active HBMs. With active musculature, the models can be used to predict kinematic response in evasive maneuvers (Kato et al., 2018; Devane et al., 2019; Larsson et al., 2019; Martynenko et al., 2019).

Typically, these active HBMs employ feedback control to activate the muscles. GHBMC and THUMS use a similar feedback loop, where neck muscles are activated based on head rotations relative to thorax rotations in relation to a reference posture, and lumbar muscles are activated based on thorax rotations relative to pelvis rotations in relation to a reference posture (Kato et al., 2017; Devane et al., 2019). Another THUMS version, THUMS-D activates the individual muscles in response to the individual muscle lengthening (Martynenko et al., 2019; Wochner et al., 2022). The SAFER HBM, when modelling a passenger, activates the neck muscles based on change in a link angle between head and T1 vertebral body, from reference posture to current posture, and lumbar muscles in the same manner for a link angle between sacrum and T10 vertebral body (Larsson et al., 2019). These active HBMs have been validated using volunteer responses in evasive maneuvers (Kato et al., 2018; Devane et al., 2019; Larsson et al., 2019; Martynenko et al., 2019; Wochner et al., 2022).

Many studies have presented volunteer evasive maneuver average responses (Ejima et al., 2012; Van Rooij et al., 2013; Ólafsdóttir et al., 2013; Kirschbichler et al., 2014; Holt et al., 2020; Chan et al., 2022). Some studies have investigated correlations between occupant kinematics and gross physical characteristics (sex, stature, BMI or age), in terms of peak displacement (Ólafsdóttir et al., 2013; Kirschbichler et al., 2014; Chan et al., 2021; Chan et al., 2022), with mixed results. For example, no significant correlation between sex and peak displacement was found in a study evaluating the effect of braking on occupant kinematics (Ólafsdóttir et al., 2013), while in another study including braking and lane change, correlation between sex and peak forward displacement was only found for braking (Kirschbichler et al., 2014). In another study with relaxed and braced volunteers (Chan et al., 2021; Chan et al., 2022), differences between average-sized males and small females were found for relaxed volunteers in low-speed frontal impacts at two acceleration levels, while in frontal-oblique low-speed impacts, no differences were found at the lower acceleration level.

In some studies, regression models have been used to predict volunteer responses based on selected characteristics (Reed et al., 2018; Reed et al., 2021; Larsson et al., 2022a), but these characteristics explain no or very little of the variation. In one study, BMI and age were significant predictors of head displacement in braking (Reed et al., 2018). It was noted that although BMI and age could predict some of the differences in displacement, considerable variance remained after accounting for passenger characteristics. In another study, the time history of passenger head forward displacement in braking could be predicted by occupant age and stature (Reed et al., 2021). It was also here noted that although age and stature could predict some of the displacement, the effect was small compared to the remaining variation not attributed to these characteristics. In a third study, sex, age, BMI, and stature were investigated as possible predictors of passenger head and torso time series displacement in five different vehicle maneuvers (Larsson et al., 2022a). All the investigated characteristics could predict some of the variances for some of the displacements. However, the effect was small compared to the effect from changing the belt system and the residual variability.

It has been suggested that the residual variability (not explained by statistical models) seen in volunteer tests could originate from voluntary movement (Reed et al., 2018; Larsson et al., 2022a). However, potentially, the variability could also stem from something other than these voluntary action or gross physical characteristics but still be related to some anatomical, physiological, or biomechanical characteristics not yet accounted for in the existing studies (Reed et al., 2018; Reed et al., 2021; Larsson et al., 2022a). For instance (Wochner et al., 2022), suggest body shape, degeneration, and fitness as potentially influential characteristics.

Some studies have investigated the effect of some of these potentially influential characteristics, using simulations with HBMs and physical tests with volunteers. For instance, posture was influential in simulations of occupant response to braking (Erlinger et al., 2022). In a test with volunteers, posture was found to influence volunteer posture stabilization in vibrational loading (Mirakhorlo et al., 2022). As a step towards modelling an elderly population, neural delay and muscle peak maximum force were identified as influencing steering wheel and brake pedal force, in simulations of bracing (Banik et al., 2021). Although the effect of posture change, muscle force and neural delay have been studied previously, neither of these studies has investigated the effect from multiple human characteristics on occupant response to evasive maneuvers, with distributions of each characteristic based on variations within a population.

Thus, the aim of this study was to investigate the sensitivity of the SAFER HBM to selected human characteristics, not yet accounted for in analysis of data from volunteer studies, in simulations of braking maneuvers, as a step towards explaining the large variation found in volunteer kinematics in these maneuvers.

## 2 Materials and methods

In this simulation study, parameters of the SAFER HBM v10.0 (Pipkorn et al., 2021) were varied to investigate the model sensitivity to these parameters, and to study how much of the variation observed in volunteer responses that can be explained by these variations. All simulations were performed with LS-DYNA MPP R12.0.0 Double Precision (SVN version 148,978, LST, Livermore, CA, United States of America). Pre-processing was done in ANSA v22, post-processing was done in MATLAB R2022a (The Mathworks Inc., Natick, MA, US), LS-PrePost V4.9 (LST, Livermore, CA, United States of America) and META v22 (BETA CAE Systems, Switzerland).

### 2.1 Passive validation

To determine the bio-fidelity of the SAFER HBM prior to parameter variations, the passive model (without any of the described updates) was validated in 4 g sled tests, by comparing model kinematics and belt forces to kinematics and belt forces recorded in two physical post-mortem human subject (PMHS) tests (Lopez-Valdes et al., 2017). The simulation setup has previously been described in (Larsson, 2020). The rigid seat was modelled with rigid material, and a compliant 3-point seat belt (0.7% strain at 1 kN) was used. The simulation was divided in two phases: gravity settling (300 ms) and acceleration phase (300 ms). Belt slack was removed during gravity settling. During gravity settling and initial phase of acceleration, the head was kept upright with 4 linear springs representing the tape used to keep the head upright in the physical tests. These springs were released from the head after 380 ms total simulation time. During gravity settling, the T1 vertebra was also constrained. The initial posture of the HBM was based on the average initial posture from the PMHS tests, and the HBM was positioned in a separate simulation, using the Marionette method, where pre-tensioned cables are used to pull selected body parts into the desired position. The rigid seat was included in the positioning simulation, and the resulting stresses of the thighs and buttock soft tissues were included as initial stresses in the validation simulation.

The kinematics and seat belt forces of the simulations were compared to the individual results from the two PMHS (referred to as PMHS1 and PMHS2) using CORA (Thunert, 2017) with settings described in Supplementary Table S3, and with visual comparison of sagittal plane kinematics and selected seat, seat belt and feet force time-histories. CORA is a software that is used to compare time-histories of (for instance) simulations to physical tests and provides a rating of the similarity between the simulation and physical test time histories. Two identical curves give a score of 1, and completely dissimilar time-histories result in a score close to 0.

In addition to the passive low-speed validation performed in this study, the stiffness of SAFER HBM cervical and lumbar spines have been validated using quasi-static flexion-extension rotation tests using functional spine units (L2-L3, C4-C5), and on the upper cervical spine (C0-C2) (Östh et al., 2020).

### 2.2 Simulation setup

Simulations of braking tests using a standard inertia reel seat belt system from (Larsson et al., 2022a) were used for the sensitivity study. In that particular test setup, initially presented in (Ghaffari et al., 2018), volunteers were seated in the front row passenger seat of a Volvo V60 and exposed to a 10 m/s^2^ braking pulse with a duration of approximately 1.3 s, Supplementary Figure S1.

The seat and restraint system models were models of a V60 seat (Östh et al., 2012), previously used in simulations of lane change maneuvers from the same volunteer test series (Larsson et al., 2019). Before all simulations, the HBM was positioned as close as possible (without introducing penetrations) to the seat cushion and seat back. Before acceleration onset, the model was exposed to gravity only during 400 ms to settle the HBM in the seat. During this settling, the controllers were initialized, with reference position for head and torso set at 250 ms. To remove belt slack during gravity settling, the belt was pre-tensioned with 6 N (modelled with a retractor element with 6 N at 0 pull-out) and then locked after 250 ms. The arms were constrained to the thighs with a pre-tensioned cable (10 N per arm).

HBM head and torso kinematics were compared to responses created from regression functions for a 45-year-old male with a stature of 175 cm, a BMI of 25 kg/m^2^ (Larsson et al., 2022a), corresponding to the SAFER HBM. Kinematics are presented in a vehicle-fixed coordinate system with positive x-axis in the vehicle forward direction and the positive *z*-axis in the downward vertical direction.

### 2.3 Sensitivity analysis

To evaluate the sensitivity of the model response to variations in human characteristics (in connection with the sensitivity analysis, these characteristics are also referred to as parameters), the multiplicative dimensional reduction method (M-DRM) presented by (Zhang and Pandey, 2014) was used. This method has been adopted in several similar studies previously (Naseri and Johansson, 2018; Naseri et al., 2020; Larsson et al., 2022b; Brynskog et al., 2022; Larsson et al., 2023). In short, a model output Y , depending on input parameters $X={\left[X_{1},\ldots ,X_{n}\right]}^{T}$ , can be described through some function, $Y=h\left(X\right)$ . The function h is approximated with reference to a fixed input point (cut-point) with coordinates c . When using the M-DRM method, the function is approximated for one of the parameters at the time, with the other parameters kept at their cut-point, Equation 1.

$$h\left(x\right)\approx h_{0}^{1-n}\prod_{i=1}^{n}h\left(x_{i},c_{-i}\right)$$ (1)

Equation 1

The mean and mean square ( $\rho_{i}$ and $\theta_{i}$ ) can then be approximated using one-dimensional integrals, computed numerically with Gaussian quadrature, Equation 2. $w_{\text{ij}}$ describes the Gauss weight for the i :th parameter and j :th Gauss point.

$$\left\{\begin{array}{c} \rho_{i}\approx \sum_{j=1}^{N}w_{\text{ij}}h\left(X_{i}^{j},C_{-i}\right)\\ \theta_{i}\approx \sum_{j=1}^{N}w_{\text{ij}}{\left[h\left(X_{i}^{j},C_{-i}\right)\right]}^{2} \end{array}\right.$$ (2)

Equation 2

Using the approximative mean and mean square ( $\rho_{i}$ and $\theta_{i}$ ), the primary sensitivity of the model to the selected parameter can be approximated according to Equation 3.

Si≈θiρi2−1∏k=1nθkρk2−1 (3)

Equation 3

With this approach, the number of simulations needed to evaluate the sensitivity of the model to n parameters, with N Gauss points becomes at most nN . If the nominal model is the same for all parameters, this is reduced to n (N-1) + 1. In this study, 7 parameters were evaluated using 5 Gauss points, resulting in 29 simulations.

### 2.4 Nominal model

**FIGURE 1:**
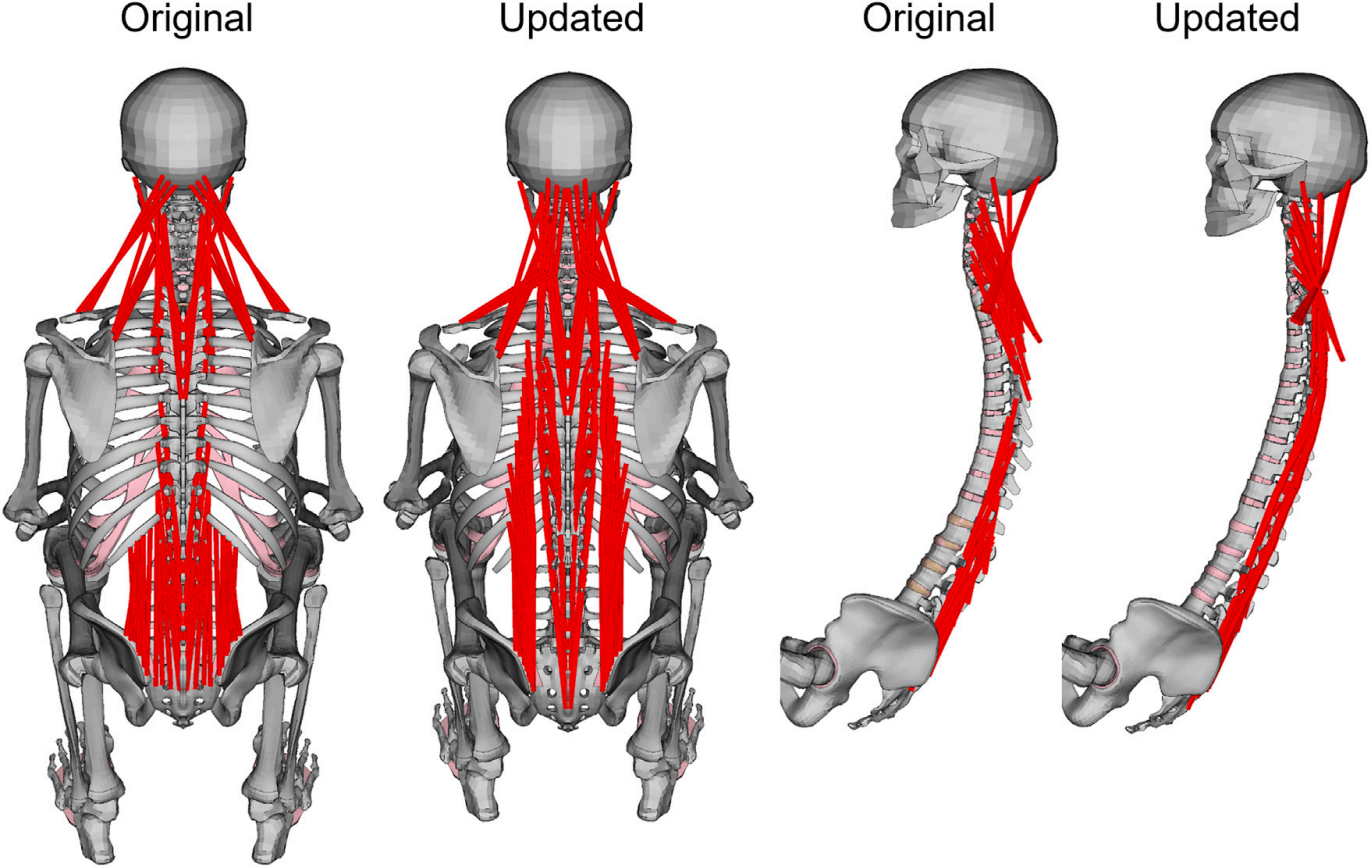
Original and updated SAFER HBM skeleton. For visibility, upper extremity and chest were removed in the side view. Muscles that were rerouted are shown in red.

The nominal model was based on the SAFER HBM v10.0 (Pipkorn et al., 2021), with updates to the spine curvature, muscle routing, and properties of the material models representing muscle and adipose tissue. The spine was aligned to the average spine curvature from (Izumiyama et al., 2018; Nishida et al., 2020), see details below. Several posterior muscles were rerouted to ensure that all extensors remained extensors through the duration of the braking, Figure 1. Without this update, combined with the updated spine curvatures used in this study, the extensors became flexors when the head and torso started to curve during braking, and the model was not able to return to upright during the maneuver. For a full description of muscle rerouting, see Supplementary Table S1. The muscle soft tissue material model properties were updated to the properties from (Lanzl et al., 2021). The adipose tissue material model properties were updated as described in the subsection Soft tissue material properties. The entire HBM was rotated 4.5° backwards around the hip (sacrum center of gravity node location) to align the HBM with the seat back.

In SAFER HBM v10.0, the skin was modelled using anisotropic material (Manschot and Brakkee, 1986) with material directions based on skin tension lines (Langer’s lines) (McIntosh and Fyfe, 2013).

Six HBM characteristics were varied; two spinal alignment parameters (see details in the section below), a neural delay parameter, a muscle physical cross-sectional area (PCSA) parameter, an adipose tissue material property parameter, a passive muscle tissue material property parameter, and a skin material stiffness parameter, with distributions according to Table 1. The process of obtaining distributions for each of these parameters is described in the sections below. Evaluation points are presented in Supplementary Table S2.

**TABLE 1:** Summary of references and assumed distributions for all parameter variations.

Parameter	References	Distribution
Spinal alignment	Izumiyama et al. (2018), Nishida et al. (2020)	Normal
Neural delay	Foust et al. (1973), Siegmund et al. (2003), Ertl et al. (2017)	Normal
Muscle PCSA	Savage et al. (1991), Frantz Pressler et al. (2006), Kamaz et al. (2007), Fortin et al. (2015)	Normal
Adipose material model properties	Gefen and Haberman (2007), Geerligs et al. (2008), Comley and Fleck (2012)	Uniform
Muscle material model properties	Van Sligtenhorst et al. (2006), Böl et al. (2012), Mohammadkhah et al. (2016), Lanzl et al. (2021)	Uniform
Skin material properties	Manschot (1985)	Lognormal (integral calculation) Normal (along material parameter variation), Lognormal (across material parameter variation)

#### 2.4.1 Spinal alignment

**FIGURE 2:**
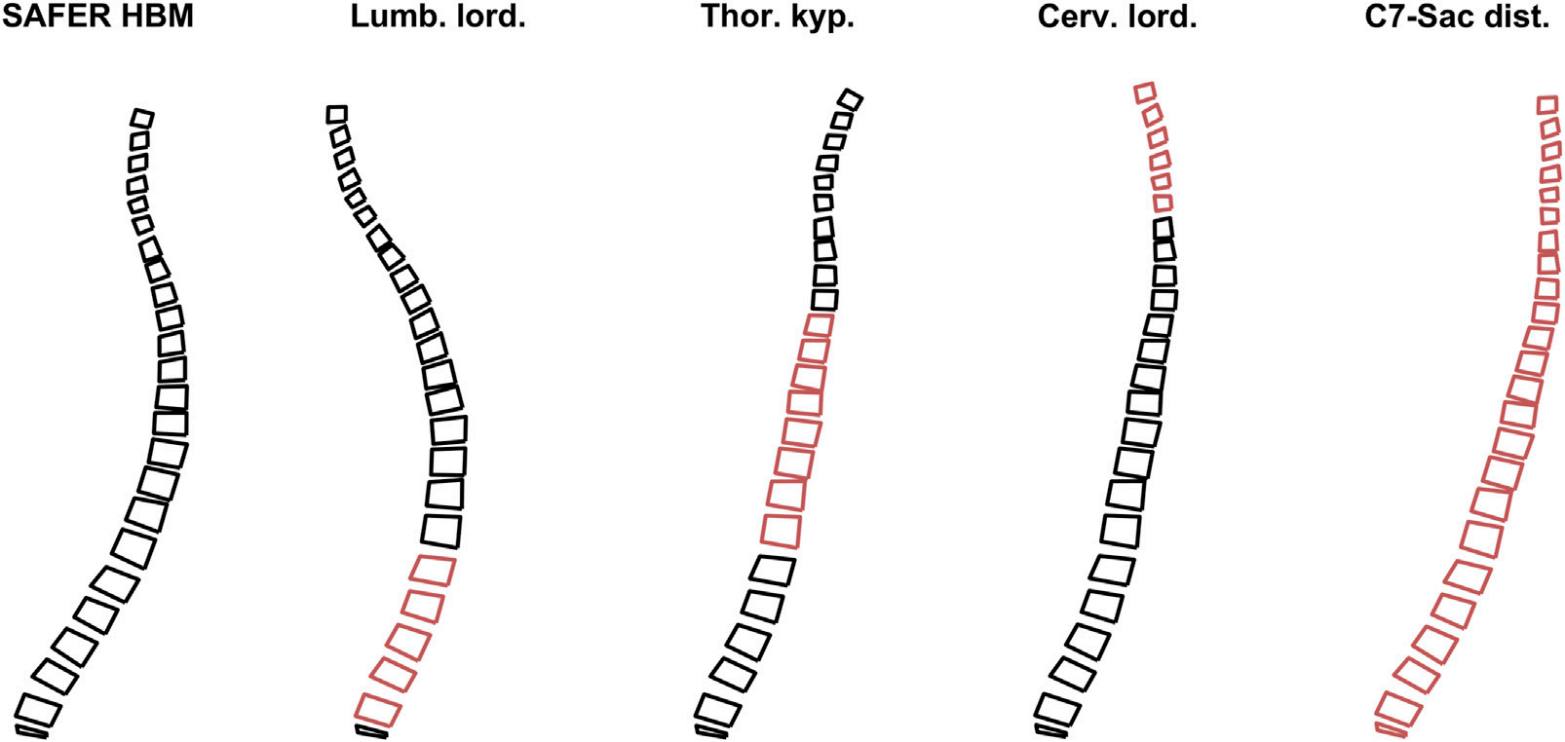
Rotation process, from original spine from SAFER HBM to the left, to subject specific spine to the right. The red vertebrae highlight the segments under alignment.

The spinal alignment is based on spinal alignment data from an x-ray study with occupants in a vehicle seat (Izumiyama et al., 2018; Nishida et al., 2020). In that study, seven measurements of spinal geometry were reported, of which 4 were spinal segment angles. In the current study, three spinal segment angles and one distance were used to define the spinal curvature. The three angles were lumbar lordosis, defined as the angle between superior vertebral endplate of L1 vertebra and inferior endplate of L5 vertebra, thoracic kyphosis, defined as the angle between superior endplate of T5 vertebra and inferior endplate of T12 vertebra, and cervical lordosis, defined as the angle between the inferior endplate of C2 vertebra and inferior endplate of C7 vertebra. The horizontal distance between C7 vertebra and sacrum was used to rotate the aligned spine in the global frame. These 4 measurements, on individual level, for the males with a BMI between 18 and 35 (a total of 36 volunteers met this inclusion criterion, average stature 171.5 cm (standard deviation (SD) 4.7 cm), average age 45 years (SD 13 years)), were transformed to vertebral positions using the procedure below (in MATLAB), visualized in Figure 2.

1. Nodal positions from 5 nodes per vertebra body were extracted from the SAFER HBM and imported in MATLAB, the 4 corner nodes in the sagittal plane (most inferior-posterior, inferior-anterior, superior-posterior, and superior-anterior points of the vertebral body, in the sagittal plane), and one measurement node (at approximately center of gravity).
2. The difference between current and target lumbar lordosis angle was calculated based on the angle difference between two vectors: one between inferior points of L5 vertebra and one between the superior points of L1 vertebra. Each joint in the segment was rotated individually, starting from L4-L5 and going upwards to L1-L2, one joint at the time. During rotation, all vertebrae above the joint were rotated rigidly together around the joint, while all vertebrae below the joint remained in the current position. The rotation was performed with a rotation matrix, around the instantaneous axis of rotation for that specific joint, based on vertebra type and size (White and Panjabi, 1978) and current vertebra position. Rotation magnitude was determined by dividing the difference between target and current segment angle with the number of joints to rotate in that segment (e.g., an 8-degree difference between target and current segment angles meant that each individual joint was rotated 2°, because there were 4 joints in the lumbar lordosis segment). This process was iterated until the target segment angle was achieved.
3. The process described in step 2 was repeated for thoracic kyphosis.
4. The process described in step 2 was repeated for cervical lordosis.
5. The whole spine was rotated rigidly around the sacrum to match the horizontal distance between sacrum and C7 vertebra.

**FIGURE 3:**
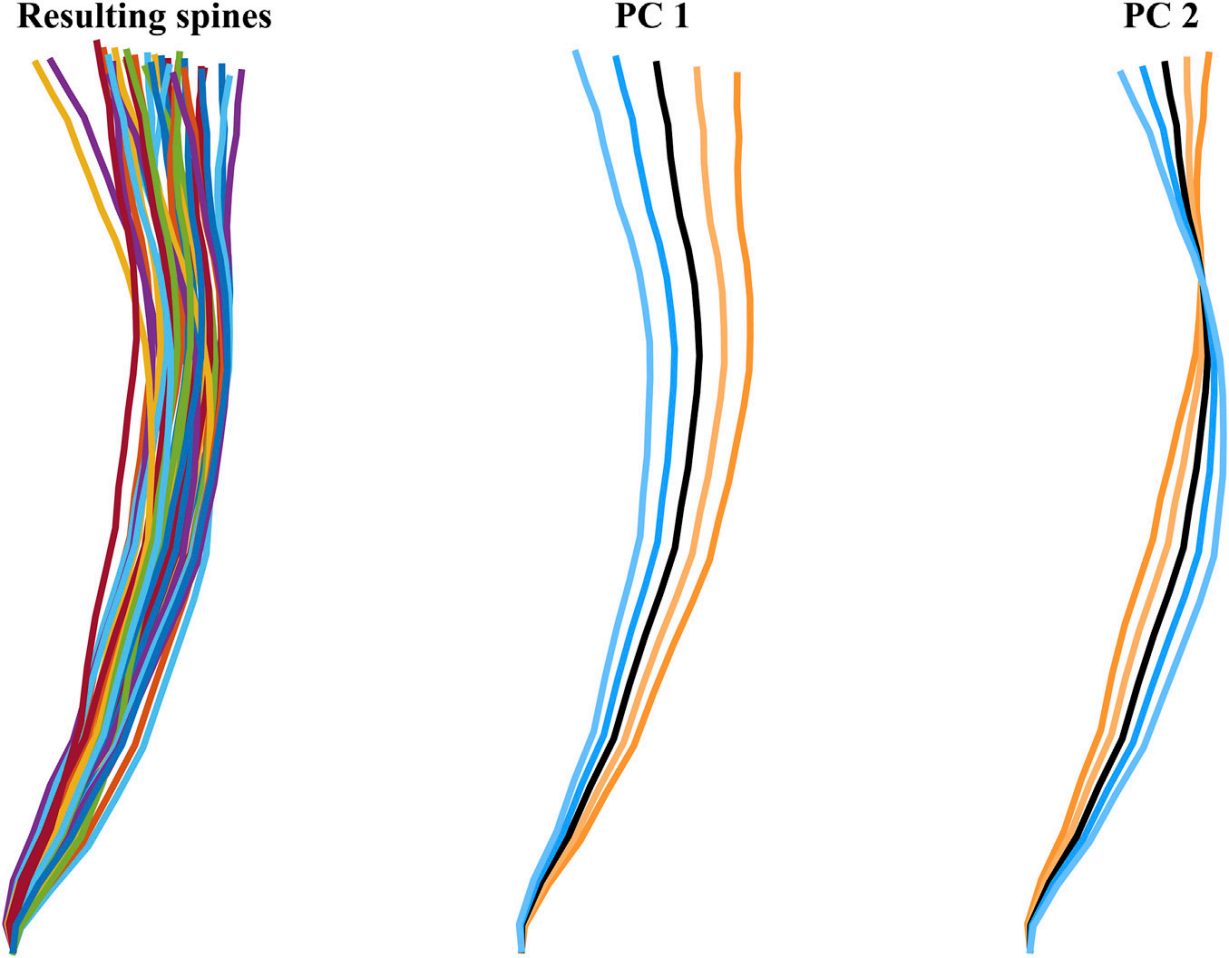
The 36 resulting spines (colorful) to the left, and the two first PCs, middle and right figures. The blue lines indicate positive SD direction, at 1 and 2 SD from average (black), the orange lines indicate negative SD direction, at −1 and −2 SD from average (black).

Using the vertical and horizontal positions of the 36 aligned spines measurement nodes (at vertebrae center of gravity), principal component analysis (PCA) (Jolliffe and Cadima, 2016; James et al., 2021) was used to find the most important variations in spinal alignment, Figure 3. For HBM spinal alignment, the two first principal components were used, Figure 3. The first PC describes a change in overall upright/reclined posture, while PC2 describes a straightening/slouching of the spine, together explaining 95% of the variance.

#### 2.4.2 Neural delay

**FIGURE 4:**
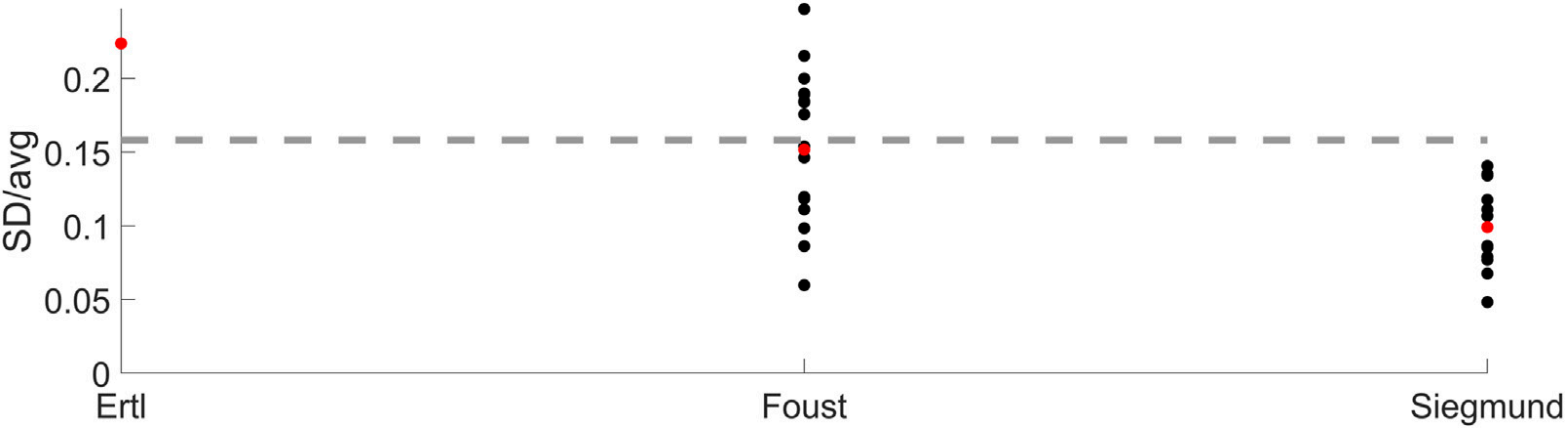
Neural delay coefficients of variation for the three experiments (Foust et al., 1973; Siegmund et al., 2003; Ertl et al., 2017). Black markers show the coefficient for each condition reported, red markers show the average per experiment and the dashed line shows the average of the averaged neural delay variation (i.e., average of red markers).

The neural delay was changed based on coefficients of variation (standard deviation divided by average) from three studies (Foust et al., 1973; Siegmund et al., 2003; Ertl et al., 2017), summarized in Figure 4. From (Ertl et al., 2017), the first component from experiment 1 was used (0.22). From (Foust et al., 1973), the average coefficient of variation from both weight drop directions, stature percentiles and age groups was calculated for male data (0.15). From (Siegmund et al., 2003) the average coefficient of variation across EMG readings from all 4 recorded neck muscles from males and all three awareness states was calculated (0.1). The (unweighted) average of the averaged coefficients of variation from each of the three experiments (0.16), Figure 4, was multiplied with the neural delays in the nominal model (20 ms for neck, 25 ms for lumbar), and those values (3.16 ms and 3.95 ms) were used as the standard deviations when varying the neck and lumbar neural delays in the simulations.

#### 2.4.3 Muscle physical cross-sectional area

**FIGURE 5:**
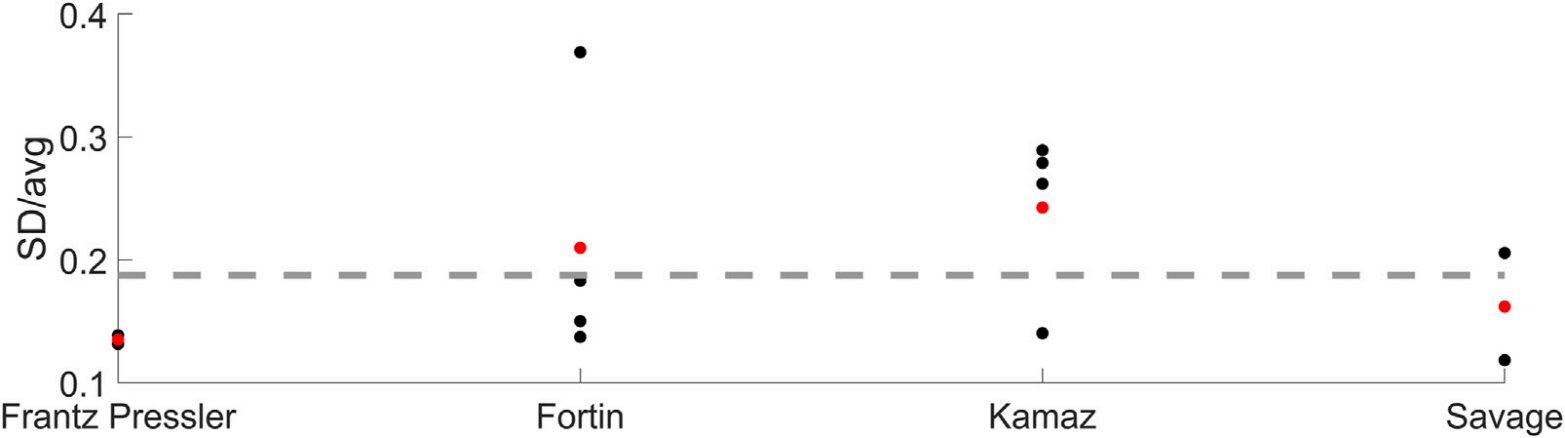
Muscle cross-sectional area coefficients of variation for the four studies (Savage et al., 1991; Frantz Pressler et al., 2006; Kamaz et al., 2007; Fortin et al., 2015). Black markers show the coefficient for each condition reported, red markers show the average per experiment and the dashed line shows the average of the averaged neural delay variation (i.e., average of red markers).

Muscle PCSA was changed based on coefficient of variation of muscle cross-sectional area (CSA) in four studies (Savage et al., 1991; Frantz Pressler et al., 2006; Kamaz et al., 2007; Fortin et al., 2015). From (Frantz Pressler et al., 2006), the average of all participants was used, and average coefficient of variation of left and right muscle was calculated (0.14). From (Fortin et al., 2015), the average coefficient of variation of all muscles and locations from baseline measurements were calculated (0.21). From (Kamaz et al., 2007), the control group was used, and the average coefficient of variation from all muscles was calculated (0.24). From (Savage et al., 1991), the group without lumbar pain was used (0.16). The (unweighted) average of the averaged coefficients of variation from each of the four experiments (0.19) was used as the SD in the parameter variation, Figure 5.

#### 2.4.4 Soft tissue material properties

**FIGURE 6:**
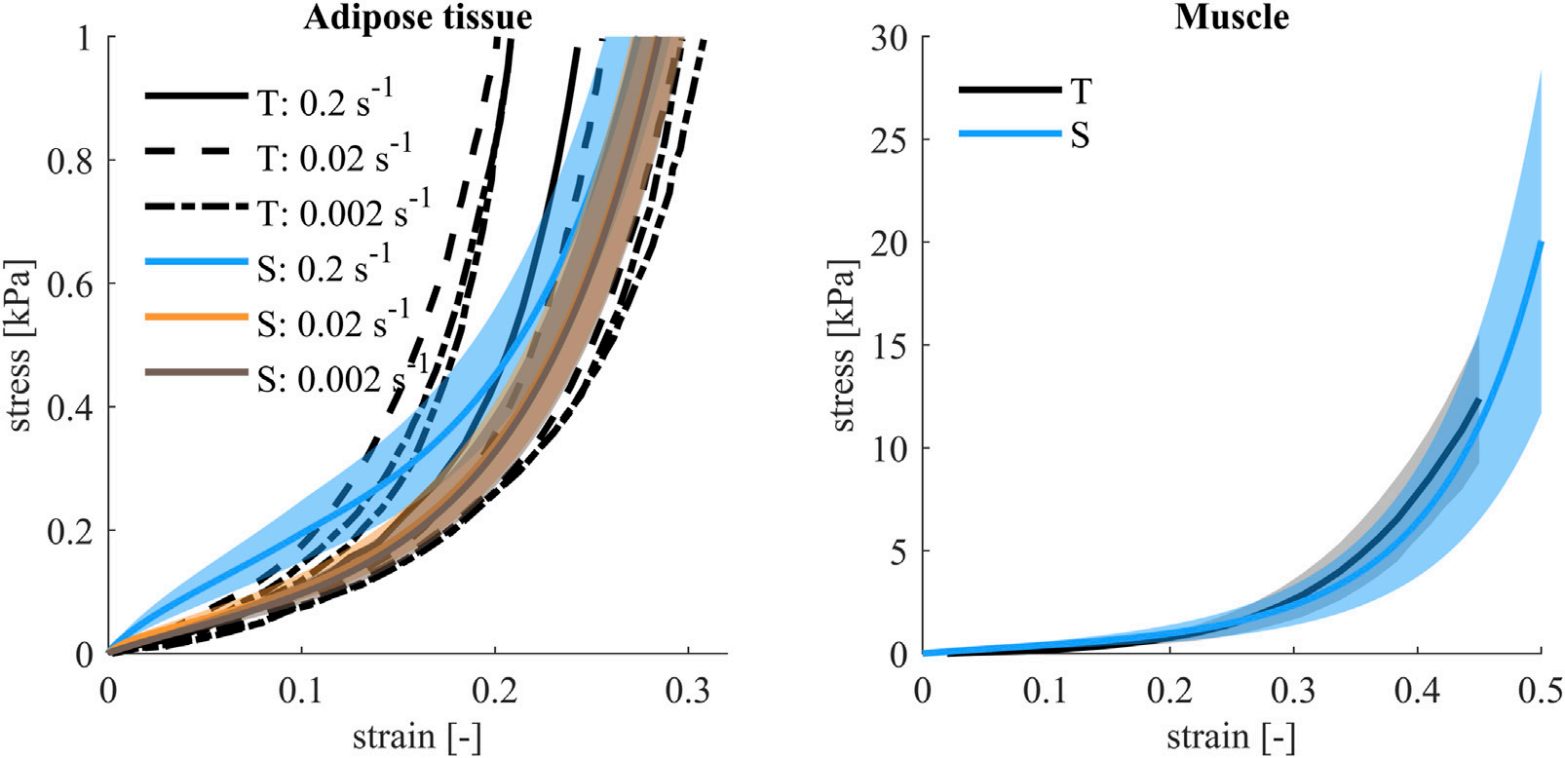
Compression stress-strain curves for adipose tissue and muscle. For adipose tissue, test (T) data (Comley and Fleck, 2012) for different strain rates was compared to simulations (S) (filled area indicates difference between maximum and minimum, nominal model plotted with a solid line) at the same strain rates as those used in testing. For muscle material, test (T) data (Böl et al., 2012), average (black) and ±1 SD (filled gray), from quasi-static cross-fiber compression tests was compared to simulations (S), (filled area indicates difference between maximum and minimum, nominal model plotted with a solid line).

Variations of soft tissue material properties were based on ranges reported in (Larsson et al., 2023). The bulk modulus of the muscle soft tissues was changed based on passive cross-fiber compressive tests (Van Sligtenhorst et al., 2006; Böl et al., 2012; Mohammadkhah et al., 2016), Table IV. The adipose tissue Poisson’s ratio, shear modulus and shear relaxation modulus were varied together. Parameter identification (Naseri and Johansson, 2018) was used to determine ranges for these properties of the material model, based on tests from (Gefen and Haberman, 2007; Geerligs et al., 2008; Comley and Fleck, 2012), Table IV. Unlike for the other parameters, adipose tissue and muscle tissue ranges were based on differences between studies, and not variations within studies. Therefore, the ranges were assigned uniform distribution in the sensitivity analysis. One-element unit cube compression tests was performed on nominal, minimum and maximum material models. Stress-strain curves were compared to those presented in (Comley and Fleck, 2012) for the adipose tissue, and those presented in (Böl et al., 2012) for the muscle. Different strain rates were simulated for the adipose tissue, Figure 6.

#### 2.4.5 Skin material properties

**FIGURE 7:**
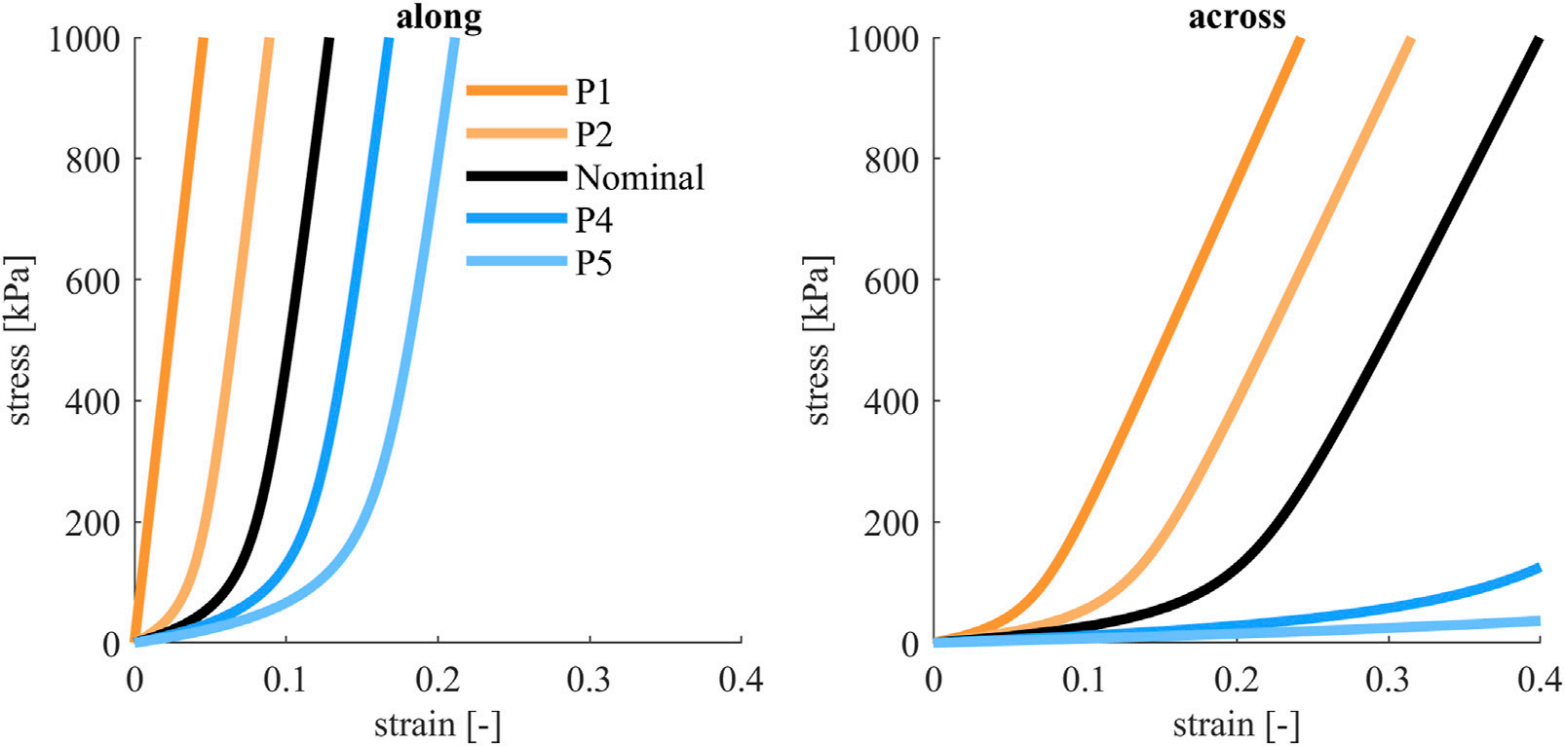
Tensile engineering stress-strain curves for skin material, along and across skin tension lines (Langer’s lines). The colored curves show the parameter variations that were evaluated, both directions were varied together. Black curves show nominal model, orange curves show stiffer models and blue show softer models.

Variations of the skin were based on median, 25th and 75th percentiles presented in (Manschot, 1985), in the material model presented in (Manschot and Brakkee, 1986). The parameter µ in the model (roughly the strain at zero stress for the tangent to linear range) was varied, the other parameters of the material model remained constant. The percentiles and median were used to fit a normal distribution to µ_along_ for the along skin tension lines direction (µ = 0.1, σ = 0.028), and a lognormal distribution for µ_across_ for the across skin tension lines direction (µ = −1.45, σ = 0.45). The evaluation points were calculated separately, but in the simulations the two parameters were varied together, with the assumption that the stiffnesses in these directions were correlated, Figure 7. A lognormal distribution was used when calculating the integral in Equation 2.

### 2.5 Simulations

**FIGURE 8:**
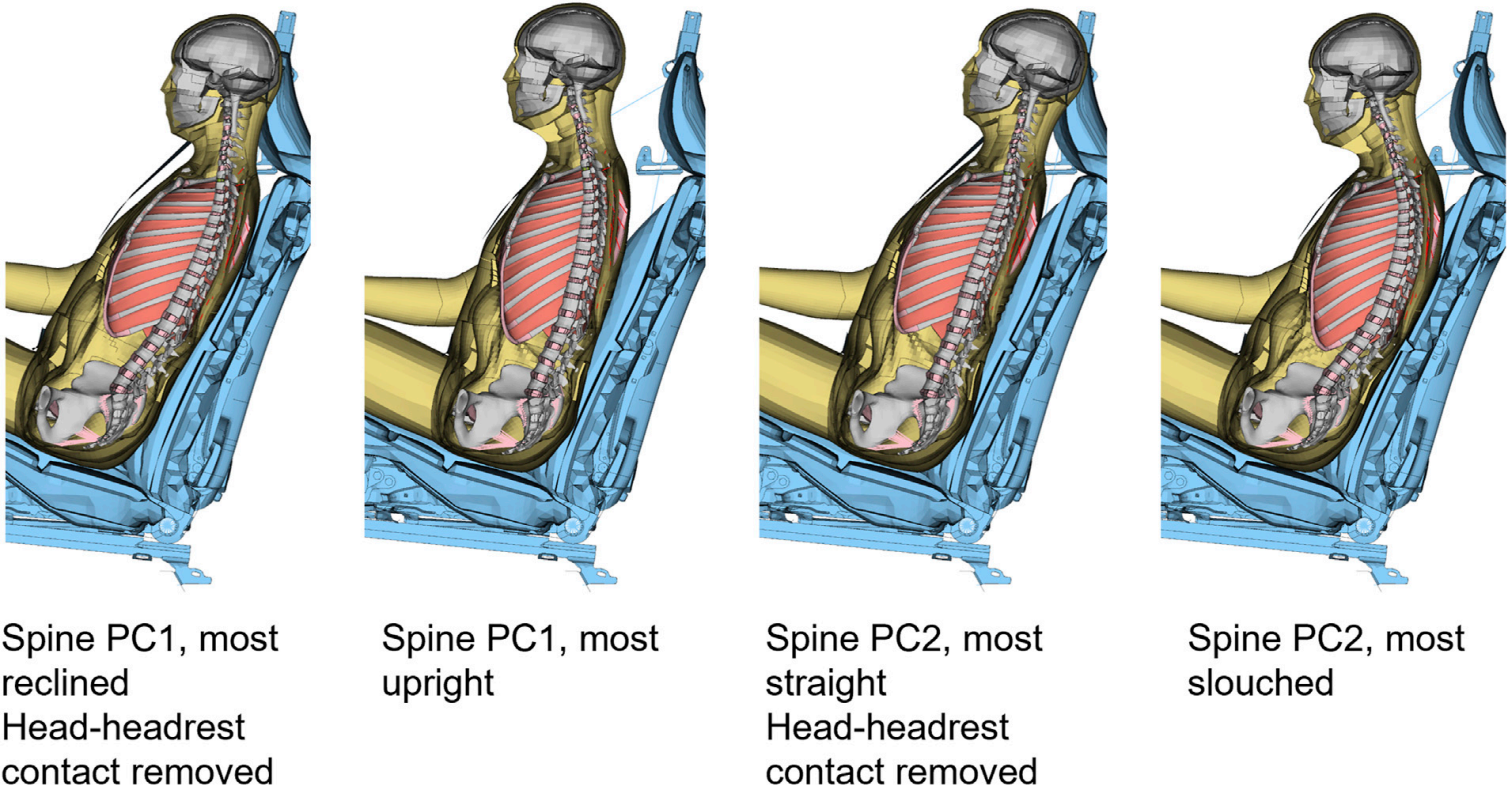
Section view of the HBM with most extreme spinal alignments, positioned in the seat, before gravity settling.

In total, 29 simulations, with variations according to Supplementary Table S2 were performed. The simulations with spinal alignment variations created some extra challenge, as the HBM had to be repositioned for each spinal curvature. In a similar process as described above, the HBM was repositioned to the desired spinal alignments during pre-simulations using the marionette method. In this method pre-tensioned cables are used to pull the model into a desired position. In the current study, cables were introduced between a node at approximately the center of gravity of each vertebra, and the desired position of that node. Nodal coordinates were exported from the final state of the pre-positioning simulations, while stresses and strains were omitted. After re-alignment the updated HBM was positioned above the seat, ready for gravity settling, using rigid translations of the model, as close as possible to the seat without penetrations between HBM and seat cushion and seat back. Because PC1 governed rotation around the hip, the rotation from the nominal model (4.5° rearwards around the sacrum center of gravity nodal position) was kept constant. If needed, the belt was rerouted to avoid penetrations between model and belt. For some of the models, the head was penetrating the headrest, and for these simulations the contact between the HBM and the headrest was removed. The HBMs with the most extreme spines, positioned above the seat (prior to gravity settling), are shown in Figure 8.

### 2.6 Comparison metrics

The sensitivity of the HBM response to change in HBM characteristics in four different metrics was investigated: peak forward displacement of the head and T1 (first peak, approximately 0.5 s into the maneuver), and average vertical displacement of head and T1. If all parameters had equal sensitivity, the sensitivity would have been 1/7, and thus a sensitivity index above 1/7 was used to identify influential parameters.

## 3 Results

The passive validation results, presented in detail in Supplementary Material, showed that the SAFER HBM v10.0 predicted the sagittal plane head displacements with good bio-fidelity, but the HBM rebounded more than the two PMHSs did.

**FIGURE 9:**
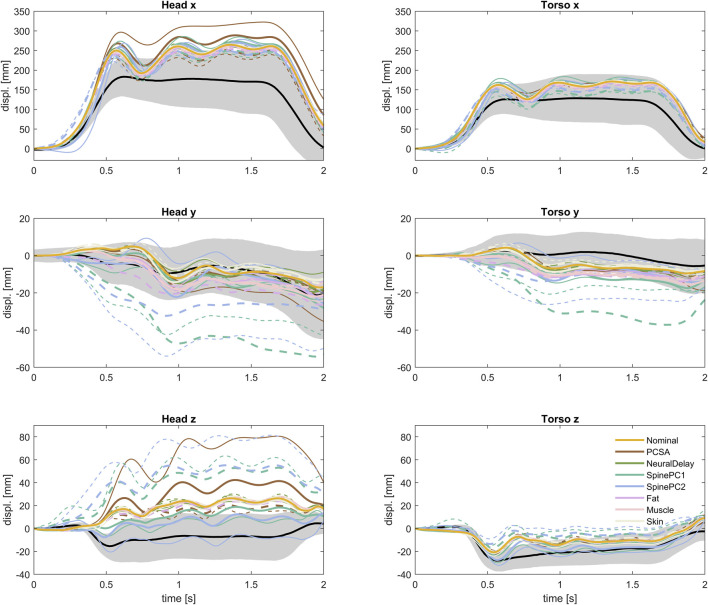
Translational kinematics of all evaluated simulation models, together with volunteer kinematics. P1 with thin solid line, P2 with thick solid line, P4 with thick dashed line, P5 with thin dashed line.

In the sensitivity analysis simulations, all simulation models predicted slightly more and slightly earlier forward displacement compared to the average volunteers, Figure 9. The predicted torso displacements were within the corridor during steady state braking (after initial peak torso excursion and before rebound), while some of the model response predictions were slightly above the corridor during loading onset and offset. Both the predicted head and torso lateral displacements were similar to the volunteer displacements. For some of the spinal alignment variations (both PC1 and PC2, P4 and P5) the predicted lateral displacements were outside the corridor. This lateral component most likely stems from the asymmetric 3-point seat belt. Comparing simulations only, the predicted head vertical displacements for simulation models differed both in magnitude and direction of displacement. Most of the models predicted a downwards head displacement, but for some of the spinal alignments (both PC1 (P1) and PC2 (P1 and P2)), the model predicted upwards head displacement for at least some of the duration, similar to most of the volunteers. The predicted torso vertical displacement was slightly smaller compared to the volunteers.

**FIGURE 10:**
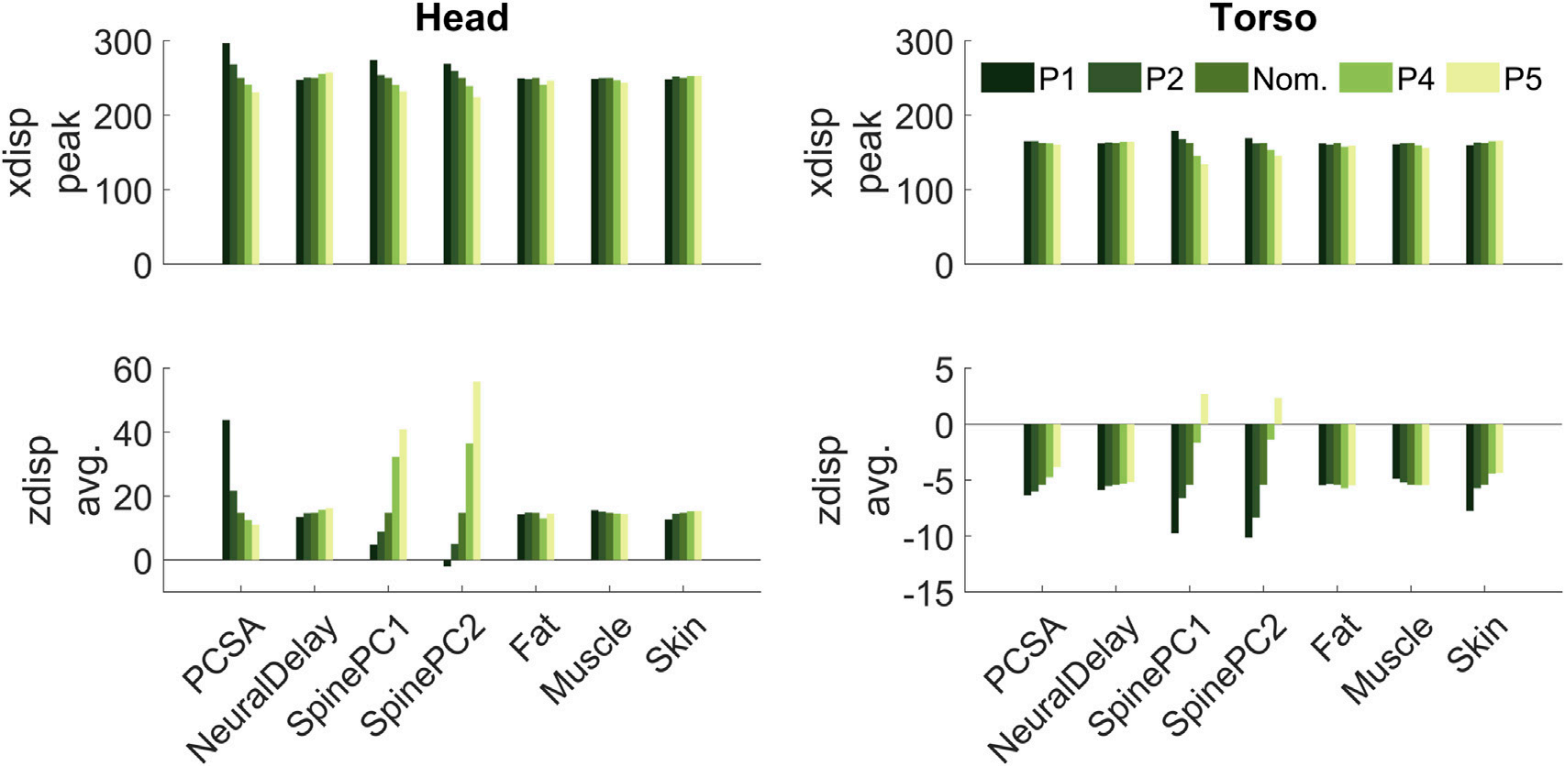
Bar plots with individual results for each of 29 simulation models in each of the 4 evaluated measures (head and torso peak forward displacement, and average vertical displacement). The nominal (Nom.) model is the same for all evaluated parameters.

Increasing muscle PCSA led to lower predicted forward displacement, both for head and torso, Figure 10, and lower predicted average vertical displacement for head and torso. The more upright spines (Spine PC1, P1 and P2, Supplementary Table S2) predicted larger peak forward displacement compared to the more reclined spines. The straighter spines (Spine PC2, P1 and P2, Supplementary Table S2) predicted larger forward displacement compared to the more curved spines. The more upright spines (Spine PC1, P1 and P2, Supplementary Table S2) predicted lower average vertical displacements compared to the more reclined spines, and the straighter spines (Spine PC2, P1 and P2, Supplementary Table S2) predicted lower average vertical displacements than the more slouched spines.

**FIGURE 11:**
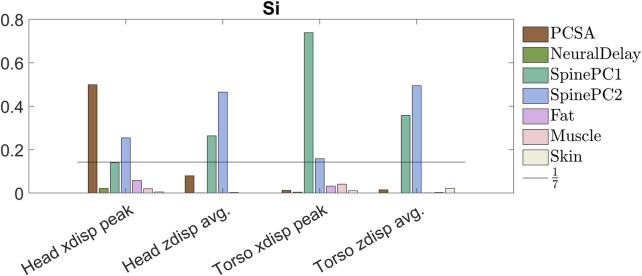
Primary sensitivity index for all compared metrics, colors indicating sensitivity of each metric to the specified parameter.

The head peak forward displacement was most sensitive to muscle PCSA, explaining around 50% of the variation, followed by spinal alignment PC2 (around 25%), and PC1 (14%), Figure 11. Head average vertical displacement was most sensitive to spinal alignment PC2 (47%) followed by spinal alignment PC1 (26%), and relatively insensitive to the other parameters. The peak forward torso displacement was most sensitive to spinal alignment PC1 (74%), followed by PC2 (16%). Torso average vertical displacement was most sensitive to spinal alignment PC2 (49%) followed by spinal alignment PC1 (36%). For all measures, the model was relatively insensitive to neural delay (maximum 2%, head peak forward displacement), fat material properties (maximum 6%, head peak forward displacement), muscle material properties (maximum 4%, torso peak forward displacement), and skin material properties (maximum 2%, average vertical displacement).

## 4 Discussion

The aim of this study was to investigate the sensitivity of the SAFER HBM to selected human characteristics, not yet accounted for in analysis of volunteer experiments, in simulations of volunteer braking maneuvers, as a first step towards explaining the large variability found in volunteer kinematics in evasive maneuvers. The sensitivity was investigated using the multiplicative dimension reduction method (M-DRM) on kinematic measures, by varying parameters for; muscle PCSA, neural delay, spinal alignment (two parameters), adipose and muscle tissue stiffness, and skin stiffness. Among the investigated parameters, spinal alignment was the most influential, influencing both occupant forward and vertical displacement, followed by muscle PCSA, influencing mainly the forward displacement.

The spinal alignment or torso posture has previously been shown to influence the occupant response in braking (Erlinger et al., 2022). The study showed that a more upright torso posture increased the peak head forward displacement, agreeing with the results from this study. Further, in the current study, spinal alignment was the most influential parameter for the head and upper torso vertical kinematics. This agrees with findings for reclined postures (Izumiyama et al., 2022), where lumbar lordosis was found influential for vertical kinematics, hypothesized to in turn affect the forward kinematics.

**FIGURE 12:**
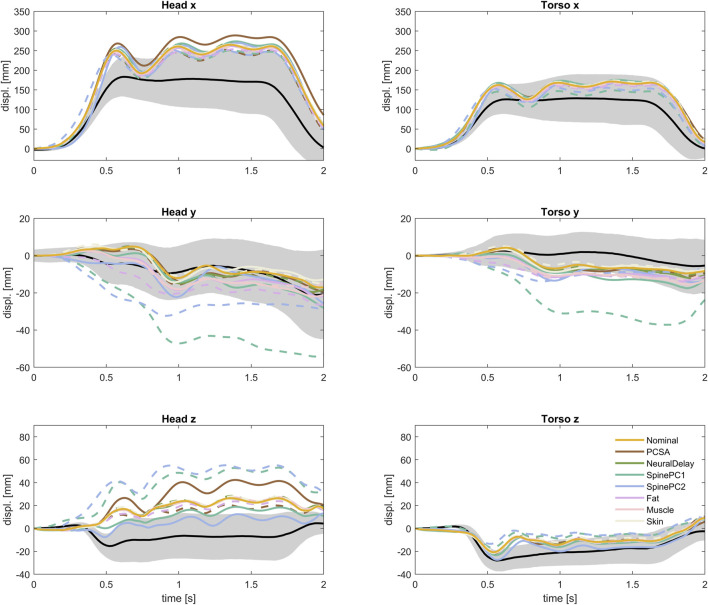
Translational kinematics of nominal model and less extreme parameter variations (P2 and P4), together with volunteer kinematics.

Showing only results from the two less extreme parameter variations, P2 and P4, Table IV, for each parameter (±1.3 SD for the normally distributed parameters), Figure 12, it was possible to compare to the volunteer response corridors (the gray area roughly corresponds to ±1 SD after accounting for occupant characteristics). The difference between the most extreme simulation models in Figure 12 were similar in size or larger than the width of the corridors for vertical displacements. For the forward displacements however, the difference between the most extreme models was smaller than the width of the corridor. Either there is a missing interaction effect, for instance between spinal alignment and PCSA, or with gross physical characteristics and the parameters varied within this study. Or, as hypothesized in previous studies, the variation in forward displacement include some voluntary component (Reed et al., 2018; Larsson et al., 2022a), or can partly be explained by variation in boundary conditions (Erlinger et al., 2022), which was not included in the current study.

**FIGURE 13:**
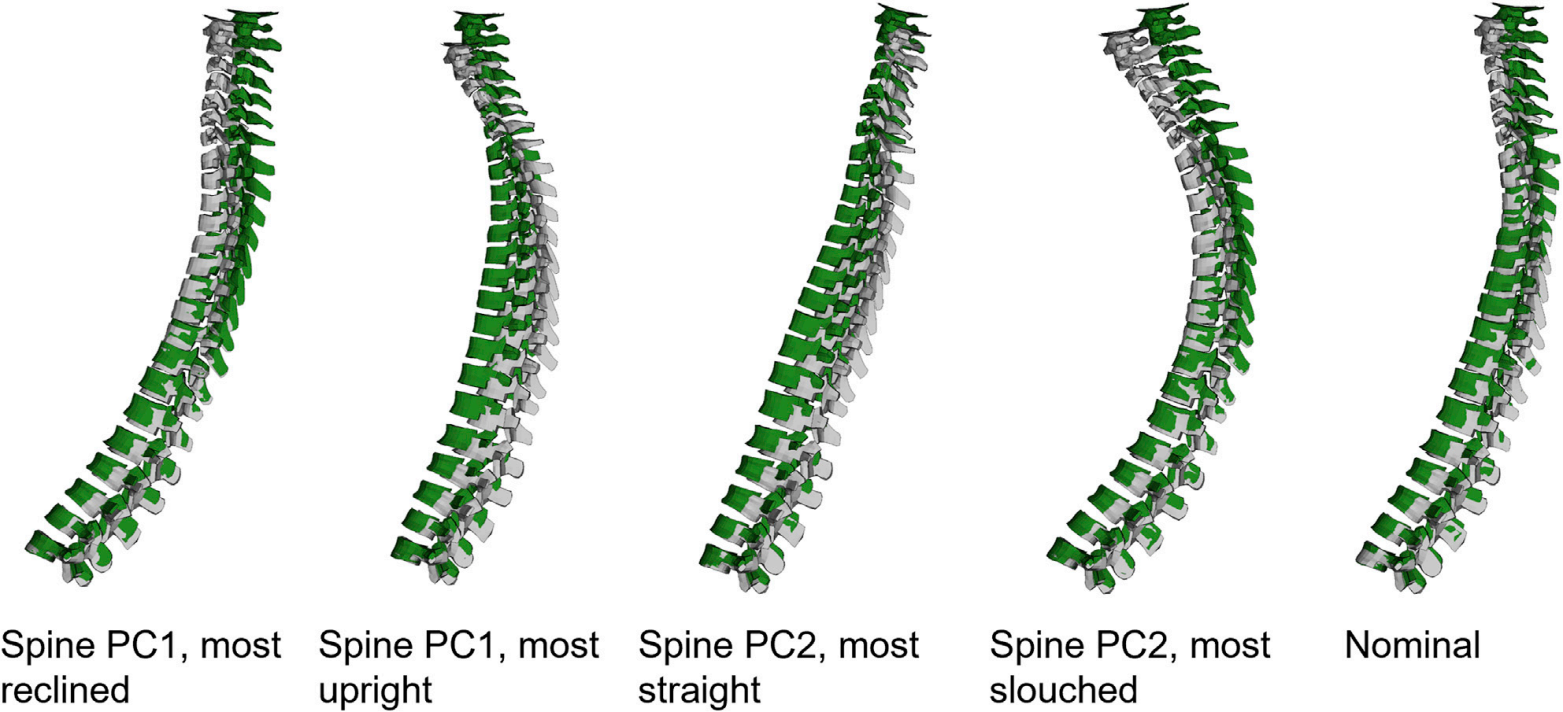
Side view of the spine of the HBM, most extreme spinal alignments and nominal model, before (green) and after (grey) gravity settling. For visualization, the models were aligned in L5 using translations only.

It should be noted that the spinal alignment was implemented prior to gravity settling. Prior to muscle controller initiation (250 ms), head and T1 were constrained in longitudinal and lateral directions, while the vertical direction was unconstrained. After controller initiation but prior to maneuver onset (250–400 ms into simulation), the posture was maintained by the controllers. Because the models were not rotated additionally after spinal alignment positioning simulations, there was some distance between the HBM and the seat for most of the spinal alignment variations, which combined with the gravity settling procedure allowed some uncontrolled re-positioning of the HBM. This led to more curved spinal alignments, in all simulations, and the difference of spinal alignment before and after gravity settling was more pronounced for models with larger horizontal distance between the head and the upper thoracic spine, Figure 13. Because the SAFER HBM typically is gravity settled prior to evasive maneuver simulations (Larsson et al., 2019; Wass et al., 2022; Östh et al., 2022), the procedure was not modified to accommodate the repositioned spines. Thus, the spinal alignment variations describe the initial posture of the HBM, and not exactly the posture of the occupant at maneuver initiation.

The spinal alignment data used for the current study is from one study, using a single seat, instead of combining different sources as was done for the other measures that were varied. This was done to include only the variation of spinal alignment seen in a single seat, but not the variation seen across many seats, thus comparable to the compared volunteer tests.

To investigate the representativeness of the spinal alignments included in the study, the average alignments reported in (Izumiyama et al., 2018) were compared to other studies where spine segmental angles were reported for seated subjects (Table 2). It should be noted that the (Sato et al., 2021) study was conducted in a rigid seat, while the other studies were conducted in vehicle seats. For the seated subjects, the average lumbar lordosis was smaller in the (Izumiyama et al., 2018) study compared to the other studies (Nam et al., 2018; Buchman-Pearle et al., 2021; Sato et al., 2021). However, the lumbar lordosis variation (SD) was similar for the compared studies (Buchman-Pearle et al., 2021; Sato et al., 2021). This difference in average lumbar lordosis, combined with the standard deviations in lumbar lordosis, means that more of the population in the (Izumiyama et al., 2018) group had more kyphotic lumbar spines compared to the other populations. Variation of lumbar lordosis was more pronounced in PC2 than in PC1. Thus, some of the spines created with PC2 variations might not be representative of other populations, for instance, the most straight PC2 spine in Figure 13. Average thoracic kyphosis was smaller for the (Izumiyama et al., 2018; Nishida et al., 2020) subjects than those in the other study (Sato et al., 2021) but with similar variation (SD). Just as for the lumbar lordosis, this indicates that the most straight spine from PC2, Figure 13, might not be representative of the population in (Sato et al., 2021). The average cervical spine curvature was similar for the (Izumiyama et al., 2018) subjects and the (Sato et al., 2021) subjects, while the variation (SD) was slightly smaller for the (Izumiyama et al., 2018) subjects compared to the (Sato et al., 2021) subjects. Thus, the cervical spines included in the study could be representative also of the (Sato et al., 2021) subjects.

**TABLE 2:** Segmental angles, seated subjects. * Values calculated from difference between upper margin of S1 to horizontal and upper margin of L1 to horizontal. ** Calculated from plots of individual measured angle.

	Seatback angle (deg)	Average lumbar lordosis (SD)	Average thoracic kyphosis (SD)	Average cervical curvature (SD)
Izumiyama et al. (2018)	23	0.9 (9.2)	20.5 (8.0)	1.3 (8.8)
Sato et al. (2021)	20	20 (12)	∼30 (10)	∼2 (15)
(Nam et al., 2018)*	23	∼10* (−)		
(Buchman-Pearle et al., 2021)**	Adjustable	6 (7)		

In this study, sensitivity was investigated using M-DRM, where only one parameter at a time was changed. This allowed for a substantially smaller simulation matrix compared to for instance Monte Carlo based methods. Although the M-DRM method provided estimates of total sensitivity (including interaction effects) as well, only primary sensitivity indexes were presented in this study, since no interactions were simulated. When using this type of sensitivity analysis, the variables are assumed to be uncorrelated (Liu et al., 2020). Since the parameters were all sampled in different studies, it is unknown if the investigated parameters correlate. One exception from this is the two spinal alignment parameters, which by design were uncorrelated since they were created from the same source and calculated using principal component analysis (James et al., 2021). For instance, aging is associated both with larger neural processing times, where a noticeable increase in reaction time was found after the age of 50 (Der and Deary, 2006), and smaller muscle CSA (Mitchell et al., 2012), and thus these parameters might potentially be correlated. A correlation between two parameters can influence the results of the sensitivity study in an unpredictable way (Caniou, 2012), since the parameters change together, while in the sensitivity study they are assumed to change independently. Since the model was insensitive to neural delay (maximum 2%), any potential effects from correlation between PCSA and neural delay would most likely also be negligible.

To provide accurate sensitivity estimates, the distributions need to represent the true distribution within the population under investigation. The method assumes that the variations in parameters all described an equally large variability, and the effect on the selected response was ranked in a relative manner. If some selected distributions indicated a larger or smaller variability compared to in the true population, the effect of that parameter might have been over- or underestimated compared to in the true population. In this study, the adipose and muscle tissue parameters were assumed to be uniformly distributed, while it is likely that the stiffness of the adipose and muscle tissues are normally or log-normally distributed within the true population. This choice of distribution might have overpredicted the effect of these two parameters, and the true effect should in reality be even smaller. For the adipose tissue, it is possible that the true variation was larger than the variation used in the study, Figure 6, since some of the curves from the tests were not within the simulated results. This could indicate that the influence from the adipose tissue was underpredicted. An increase in effect from additional variation in adipose tissue stiffness would likely not be enough to increase the sensitivity result above that of the more influential parameters, Figure 11, since the results from the more extreme variations of stiffness did not vary much from the results from the nominal model, Figure 10. The model was relatively insensitive to the passive muscle stiffness parameter, and since the distribution was similar for tests and simulations, Figure 6, the true effect of this parameter was likely negligible. Additionally, of the properties included in the study, the model was least sensitive to skin material properties. Since only one of three parameters in the skin material model were varied, it is possible that the true effect from skin stiffness is slightly larger, however since the influence from skin stiffness was smaller than all other parameters, any additional variation in the material model would most likely also result in low sensitivity.

When calculating the spinal alignments, the horizontal distance between C7 and sacrum was used to rigidly rotate the re-aligned spine. Since this distance was measured in absolute distance, the same spinal rotation of subjects with different torso height would result in different absolute distances. Since no sitting height was provided in the data, no normalization was performed before the rigid rotation. Because the stature of the SAFER HBM (175 cm) is slightly larger than the average stature in the spinal alignment data set (171 cm) there is a risk that some of the generated spinal alignments were slightly more vertical compared to how the volunteers in (Izumiyama et al., 2018) were sitting in the experiment.

### 4.1 Limitations and future work

In this study, sensitivity of displacements in braking to 7 variations of human characteristics were investigated. There are many more human characteristics, unrelated to gross characteristic or volitional control, not included in this study, that could influence the kinematics. For instance, cervical spine ligament stiffness variations were not included. The lever arm for ligament elements in bending of the cervical spine is small relative to the lever arm of the skin, fat and muscle tissue that were varied in the study, which is why these were included instead of the cervical spine ligaments. Additionally, arm posture and leg posture were not included in this study, although identified as important in a previous study (Erlinger et al., 2022).

As discussed above, the spinal alignments used in this study might not be representative of a general population or the alignments of the volunteers in the comparison data. The thoracic kyphosis was different between the study used (Izumiyama et al., 2018) and the reference (Sato et al., 2021), with larger thoracic kyphosis in the Sato study, where a rigid seat was used. Thus, it is possible that the rigid seat produced more thoracic kyphosis. In (Sato et al., 2021), cervical lordosis and thoracic kyphosis were correlated. It is possible that if the studies instead had been comparable in thoracic kyphosis, for instance if a more similar seat had been used, the subjects from the Sato study would have had more lordotic cervical spines on average, making the cervical spines used in the current study less lordotic than they would have been for another population. Sine the spinal alignment was found to be the most influential characteristic, it is of importance to use a spinal alignment that is representative of the intended occupant.

The gravity settling procedure might have affected the sensitivity results, since the posture was controlled for prior to gravity settling while modified differently during gravity settling, Figure 13. Additionally, the gravity settling could also have influenced the results for the PCSA, where the muscle size and thus muscle strength was varied, but the baseline muscle activity was unchanged. This was because the baseline muscle activity was needed to maintain the head position during gravity settling (after controllers were initiated but before acceleration onset, i.e., 250–400 ms into the simulation), a variation in muscle strength without a change in baseline activity slightly altered the gravity settling behavior, because a model with larger muscles had larger muscle forces during gravity settling compared to a model with smaller muscles.

The results for varied PCSA indicate that an occupant with larger muscles displaces less compared to an occupant with smaller muscles. With the controllers in the HBM, the assumption is that all occupants would utilize the same muscle activation strategy in terms of portion of muscle strength used, regardless of muscle size. In reality, an occupant with larger muscles might select to use less of their available muscle capacity to instead use the same muscle force level, meaning that or there would be no correlation between forward displacement and muscle size. This could be investigated in future volunteer tests by investigating correlation between occupant muscle size (or strength) and peak forward displacement and muscle activation. This could also be investigated retrospectively for volunteer tests where muscle strength, muscle activity and peak displacement has been recorded, such as (Östh et al., 2013). If there is a correlation between muscle size and peak displacement, but no correlation with muscle activity, occupants use similar strategies in terms of portion of muscles used. If there instead is a correlation between muscle size and activation level, but no correlation with peak displacement, the occupants use a similar strategy in terms of muscle force level. If both measures correlate to muscle size, the occupants with different muscle size use both a different strategy for available force and portion of muscle used. If there instead is no correlation, the muscle size is not a meaningful predictor of response to braking, and the indication of the importance in this study should be attributed to modelling assumptions alone.

Although the aim of the study was to take a step towards understanding the variability among occupant response to evasive maneuvers, only braking was included in the study. It is possible that the results do not generalize to all evasive maneuvers, and further studies including lane change are needed to understand if the same parameters that influence response to braking also influence the response to lane change.

Based on the results from this study, future tests with volunteers exposed to braking maneuvers should target measuring the spinal alignment of the volunteers, and muscle strength (or cross-sectional area of the muscles if possible), to further enhance the understanding of why the occupant response to vehicle maneuvers vary within the population.

## 5 Conclusion

In simulations of braking maneuvers, spinal alignment was shown to be most influential of the investigated parameters, followed by the muscle physical cross-sectional area. The results can be used in future tuning of the HBMs, and in design of future volunteer studies to investigate correlation between occupant characteristics and kinematics.

## Data Availability

The original contributions presented in the study are included in the article/Supplementary Material, further inquiries can be directed to the corresponding author.
