# Supplementary material for: Investigating sources for variability in volunteer kinematics in a braking maneuver, a sensitivity analysis with an active human body model
Source: Front Bioeng Biotechnol. 2023 Oct 16;11:1203959. doi: 10.3389/fbioe.2023.1203959 (PMC10614285; doi:10.3389/fbioe.2023.1203959)
Supplement: Supplementary file 1 [file DataSheet1.PDF]

Supplementary Material

Investigating sources for variation in passenger kinematics in braking maneuvers through sensitivity analysis, a simulation study.

Emma Larsson [1], Johan Iraeus [1], Johan Davidsson [1*]

[1] Department of Mechanics and Maritime Sciences, Chalmers University of Technology, Gothenburg, Sweden

*** Correspondence:**
Johan Davidsson
johan.davidsson@chalmers.se

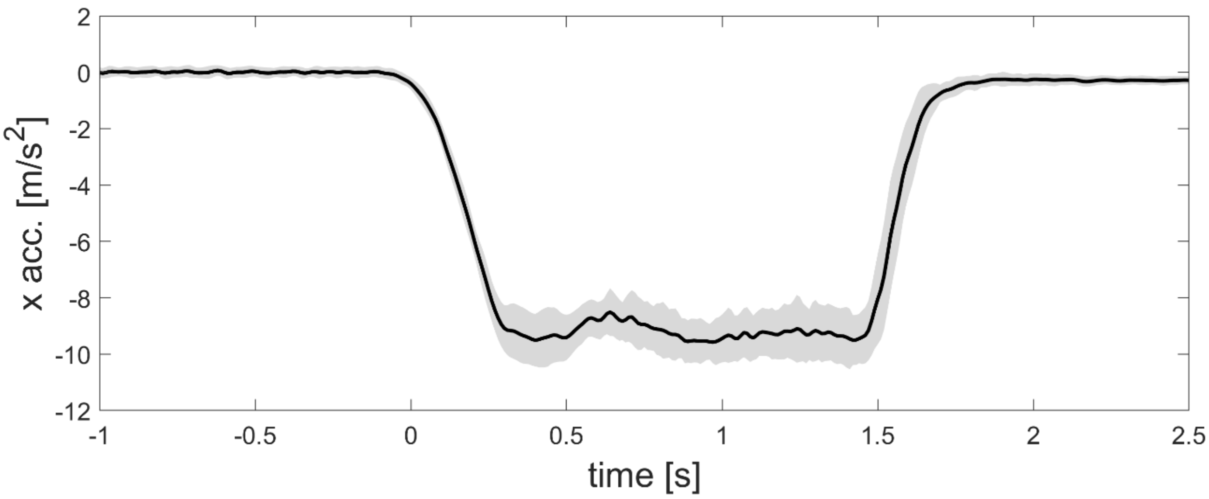

Figure S1. Average (black) and ±1 standard deviation (SD) (gray filled) longitudinal acceleration of braking maneuver (Larsson et al., 2022a).

Table S1. Muscles updated, per side

Muscle	Origin (Marieb and Hoehn, 2019)	Origin v10	Origin updated model	Insertion (Marieb and Hoehn, 2019)	Insertion v10	Insertion updated model	No. parts/elements v10	No. parts/elements updated model
Erector spinae iliocostalis lumborum	Iliac crest	Iliac crest	Iliac crest	Angles of ribs	12th rib	3rd to 12th rib	8/8	9/54
Erector spinae iliocostalis cervicis	3rd to 6th rib	4th to 6th rib	4th to 6th rib	Transverse process C4-C6	Transverse process C4-C6	Transverse process C4-C6	3/3	3/12
Erector spinae longissimus thoracis	Transverse process of vertebrae	Spinous process of vertebrae up to L2	Spinous process of vertebrae up to L3	Transverse process of vertebrae and ribs superior to origin	7th to 12th rib	5th to 12th rib, transverse process of T5 to T10	12/12	15/86
Erector spinae longissimus cervicis	Transverse process of vertebrae	Transverse process T2 to T6	Transverse process T2 to T6	Transverse process of vertebrae	Transverse process C2 to C6	Transverse process C2 to C6	5/5	5/24

one part unchanged between v10 and updated model								
Trapezius<br><br>only upper part included in model	Occipital bone, ligamentum nuchae, transverse process C7-T12	Occipital bone	Occipital bone	Acromion, spine of scapula, lateral third of clavicle	Clavicle	Clavicle	3/3	3/6
Levator scapulae	Transverse process C1-C4	Transverse process C1-C4	Transverse process C1-C4	Medial border of scapula, superior to spine	Medial border of scapula, superior to spine	Medial border of scapula, superior to spine	4/4	4/8
Splenius capitis<br><br>one part updated	Ligamentum nuchae, spinous process of C7-T6	Spinous process of C5-T3	Spinous process of C5-T3	Mastoid process of temporal bone and occipital bone	Mastoid process of temporal bone	Mastoid process of temporal bone	6/6	6/7
Splenius cervicis	Ligamentum nuchae, spinous process of C7-T6	Spinous process of T4-T6	Spinous process of T4-T6	Transverse process of C2 to C4	Transverse process of C2 to C4	Transverse process of C2 to C4	3/3	3/21

Semispinalis capitis<br><br>one part updated	Transverse process C7-T12	Transverse process C5-T3	Transverse process C5-T3	Occipital bone	Occipital bone	Occipital bone	5/5	5/6

Table S2. Simulation matrix with all parameter variations.

Parameter (Distribution, Normal (N), uniform (U), Lognormal (L))		Measure type [unit]	P1 (-2.857 SD for normal distributions)	P2 (-1.3556 SD for normal distributions)	Nominal (P3)	P4 (1.3556 SD for normal distributions)	P5 (2.857 SD for normal distributions)
PCSA (N)		Scale factor [-]	0.4572	0.7424	1	1.2576	1.5428
Neural delay (N)	Neck	Time [ms]	10.9719	15.7163	20	24.2837	29.0281
	Torso		13.7148	19.6454	25	30.3546	36.2852
Spinal alignment PC1 (N)		Nodal position (side views)	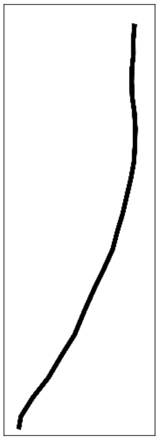	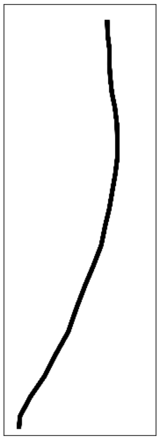	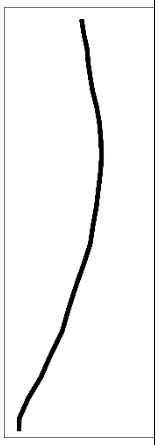	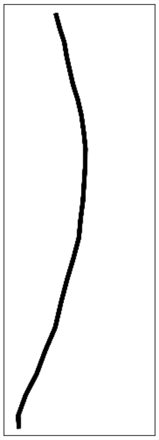	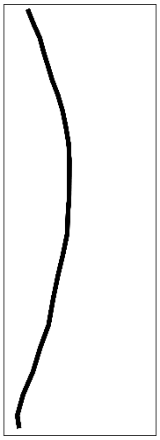
Spinal alignment PC2 (N)		Nodal position (side views)	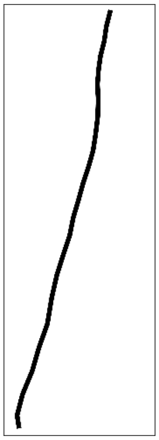	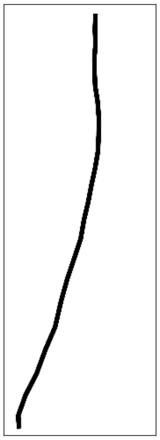	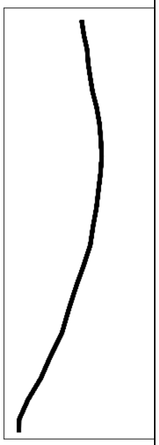	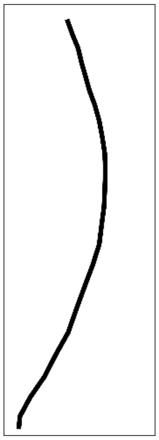	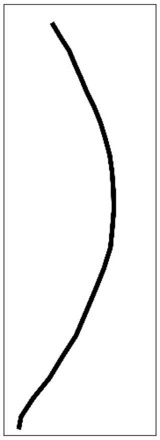

Adipose tissue<br><br>(Ogden rubber material model) (U)		PR [-]	0.499826	0.499931	0.499980	0.499992	0.499994
		MU1 [Pa]	29.6	32.0	35.5	39.0	41.4
		G1 [-*10^6]	0.58439	0.68000	0.82000	0.96000	1.05561
		G2 [-*10^6]	1.31488	1.53000	1.84500	2.16000	2.37512
		G3 [-*10^6]	1.60708	1.86999	2.25500	2.64001	2.90292
Muscle tissue (Ogden rubber material model) (U)		MU1 [Pa]	67	84	108	132	149
Skin (Anisotropic material model) (L)	Along (N)	μ	4.0000e-06	0.0420	0.0800	0.1180	0.1600
	Across (L)	μ	0.0649	0.1275	0.2	0.4317	0.8484

Table S3. CORA settings for comparison of simulation results to PMHS tests. Only correlation rating used.

	Weight	
Corridor rating	0	
Correlation rating	1	
	Shape	0.5
	Size	0.25
	Phase shift	0.25

1 Validation results

Head kinematics were predicted with good bio-fidelity compared to both PMHS test, while T1 kinematics was predicted wit poor to fair accuracy, Figure S2.

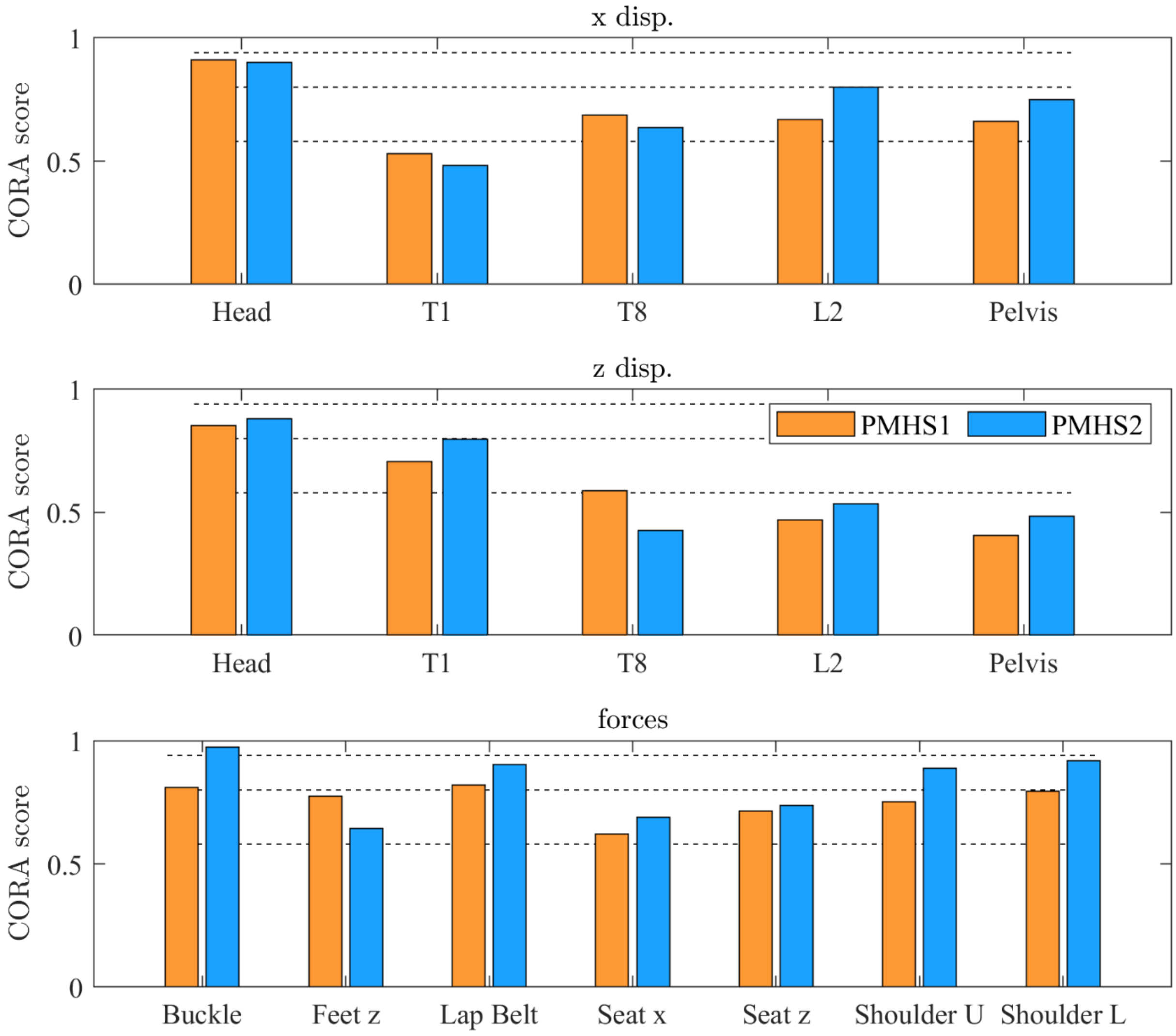

Figure S2. CORA scores for HBM compared to the two PHMS tests. The top row shows x displacement scores, middle row shows z displacement scores and the bottom row shows force scores. The dashed lines show thresholds for CORA scores (from bottom to top: poor, fair, good and excellent).

HBM head and T1 kinematics were similar in size and timing compared to in the PMHS tests, Figure S3. The HBM showed some rebound after around 20s, which was not present in the PMHS tests.

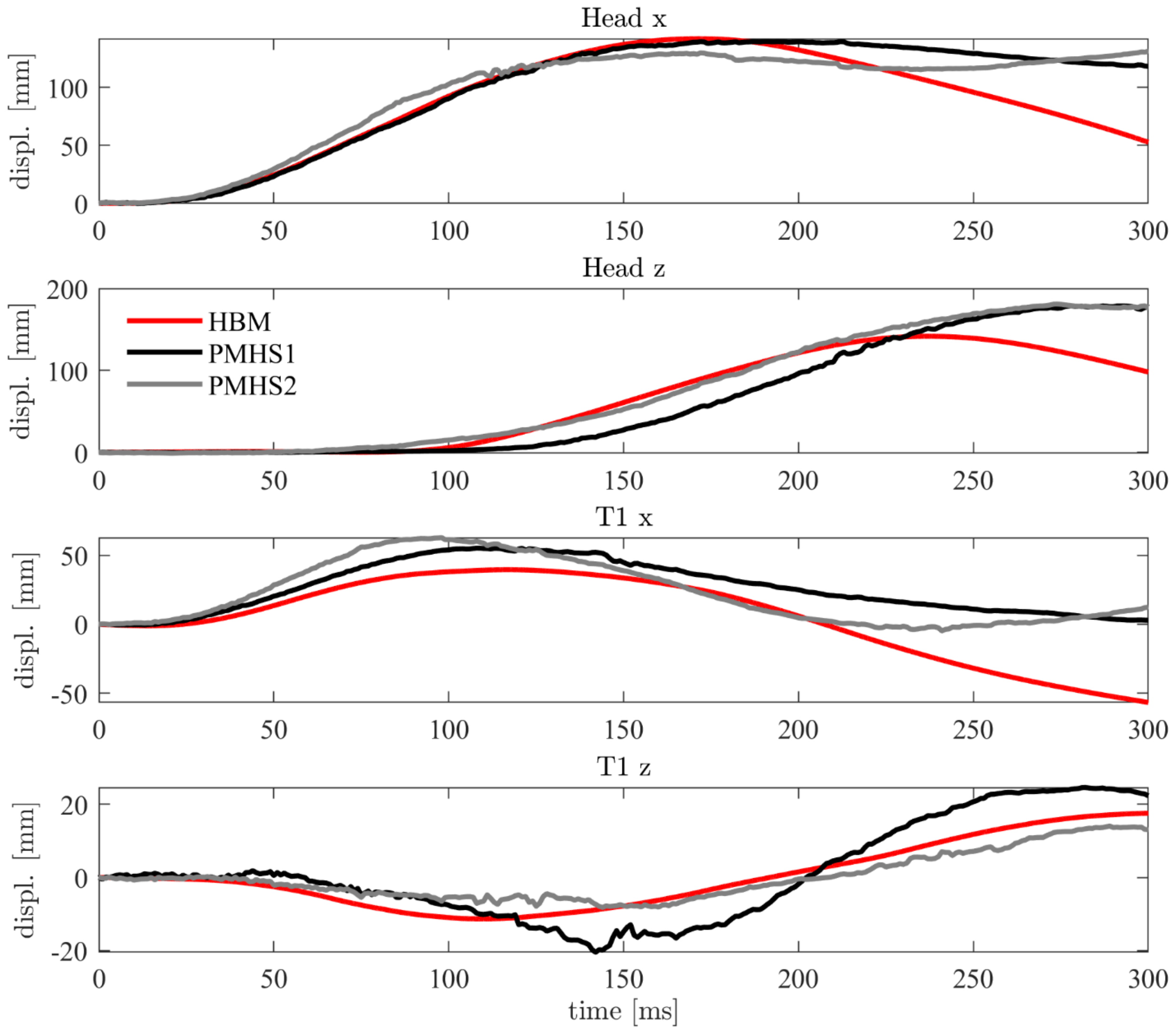

Figure S3. Head and T1 time history displacements in x and z. HBM in red, PHMS in black and grey.

The HBM rebounding was more visible when comparing sagittal plane kinematics, Figure S4.

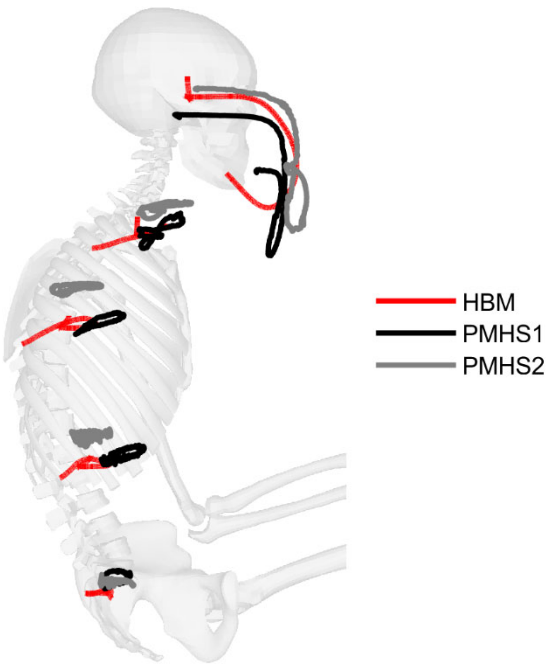

Figure S4. Full-body sagittal plane kinematics overlayed on SAFER HBM. From bottom to top: pelvis, L2, T8, T1 and head. HBM in red, PHMS in black and grey.

Belt forces were similar compared to in the test with PMHS2, but not similar to forces in the test with PMHS1, Figure S5. Feet forces and seat forces were similar to both tests.

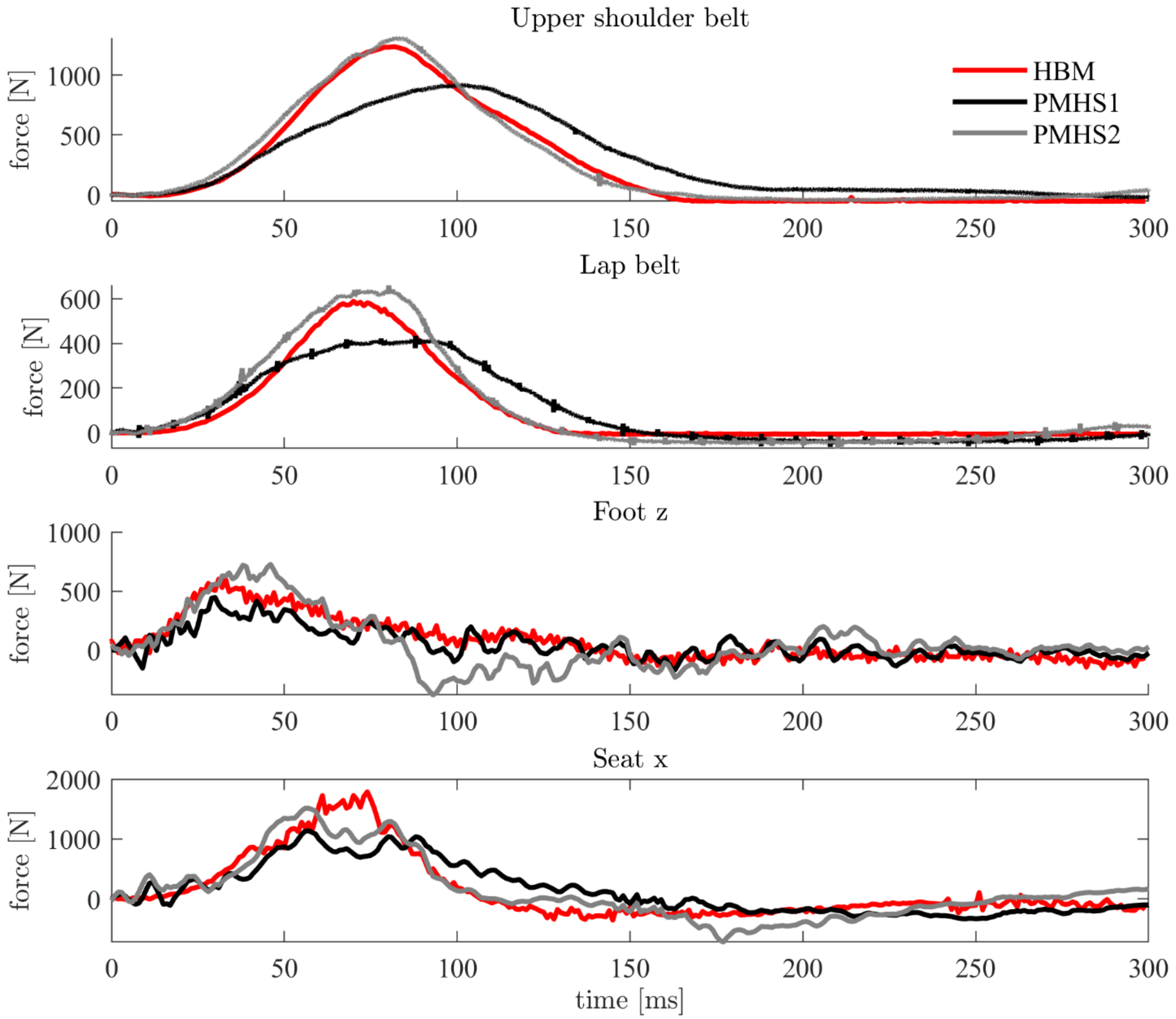

Figure S5. Belt, feet and seat force time histories. HBM in red, PHMS in black and grey.
